# Hyaluronan/Diethylaminoethyl Chitosan Polyelectrolyte Complexes as Carriers for Improved Colistin Delivery

**DOI:** 10.3390/ijms22168381

**Published:** 2021-08-04

**Authors:** Natallia V. Dubashynskaya, Sergei V. Raik, Yaroslav A. Dubrovskii, Elena V. Demyanova, Elena S. Shcherbakova, Daria N. Poshina, Anna Y. Shasherina, Yuri A. Anufrikov, Yury A. Skorik

**Affiliations:** 1 Institute of Macromolecular Compounds, Russian Academy of Sciences, Bolshoi VO 31, 199004 St. Petersburg, Russia; dubashinskaya@gmail.com (N.V.D.); raiksv@gmail.com (S.V.R.); poschin@yandex.ru (D.N.P.); 2 Almazov National Medical Research Centre, Akkuratova 2, 197341 St. Petersburg, Russia; dubrovskiy.ya@gmail.com; 3 Institute of Chemistry, St. Petersburg State University, Universitetskii 26, Peterhof, 198504 St. Petersburg, Russia; annakirillova980@mail.ru (A.Y.S.); anufrikov_yuri@mail.ru (Y.A.A.); 4 Research and Training Center of Molecular and Cellular Technologies, St. Petersburg State Chemical Pharmaceutical University, Prof. Popova 14, 197376 St. Petersburg, Russia; 5 State Research Institute of Highly Pure Biopreparations, Pudozhsakya 7, 197110 St. Petersburg, Russia; lenna_22@mail.ru (E.V.D.); elenka.shcherbakova@ya.ru (E.S.S.)

**Keywords:** colistin, polymyxin, hyaluronic acid, diethylaminoethyl chitosan, polyelectrolyte complexes, drug delivery system, ESKAPE pathogens, antimicrobial activity

## Abstract

Improving the therapeutic characteristics of antibiotics is an effective strategy for controlling the growth of multidrug-resistant Gram-negative microorganisms. The purpose of this study was to develop a colistin (CT) delivery system based on hyaluronic acid (HA) and the water-soluble cationic chitosan derivative, diethylaminoethyl chitosan (DEAECS). The CT delivery system was a polyelectrolyte complex (PEC) obtained by interpolymeric interactions between the HA polyanion and the DEAECS polycation, with simultaneous inclusion of positively charged CT molecules into the resulting complex. The developed PEC had a hydrodynamic diameter of 210–250 nm and a negative surface charge (ζ-potential = −19 mV); the encapsulation and loading efficiencies were 100 and 16.7%, respectively. The developed CT delivery systems were characterized by modified release (30–40% and 85–90% of CT released in 15 and 60 min, respectively) compared to pure CT (100% CT released in 15 min). In vitro experiments showed that the encapsulation of CT in polysaccharide carriers did not reduce its antimicrobial activity, as the minimum inhibitory concentrations against *Pseudomonas aeruginosa* of both encapsulated CT and pure CT were 1 μg/mL.

## 1. Introduction

Nanoparticles (NPs) based on natural and modified polysaccharides are well established as potential platforms for the treatment of severe infectious diseases. These drug delivery systems protect active pharmaceutical substances from untimely destruction in the body, while also providing controlled release, overcoming biological barriers, and targeting delivery of active agents to infection sites, thereby reducing drug doses and side effects [1,2,3,4,5]. In addition, natural polysaccharides (hyaluronic acid (HA), alginic acid, starch, and dextrin) and semi-synthetic chitin derivatives, including chitosan and its derivatives (e.g., diethylaminoethyl chitosan (DEAECS), succinyl chitosan, and glutaryl chitosan), are biocompatible, biodegradable, and non-toxic, while simultaneously providing suitable drug loading efficiency [6,7,8,9].

One potential drug that could benefit from complexation with NPs is the antimicrobial drug colistin (CT). CT is a mixture of the cyclic polypeptides colistin A (polymyxin E1) and colistin B (polymyxin E2), which differ in their fatty acids [10]. CT is currently used as a last-reserve drug against multidrug-resistant infections, including nosocomial pneumonia, caused by Gram-negative bacteria (e.g., *Pseudomonas aeruginosa*, *Klebsiella pneumoniae*, *Escherichia coli*). Severe systemic infections require administration of CT by injection; however, because the small CT molecules are quickly eliminated from systemic circulation, high drug doses are required. Further disadvantages of CT therapy are its side effects of neurotoxicity and nephrotoxicity [11,12,13]. These disadvantages can be reduced by using nanotechnology-based drugs [3,4,14]. 

Polyelectrolytes are widely used in biomedicine due to the variety of their conformations and nanostructures, as well as their molecular interactions with biomedical materials. For example, by varying the molar ratio between two oppositely charged polyelectrolytes, it is possible to obtain different types of supramolecular systems (such as host–guest complexes and interpolyelectrolyte complexes) through cooperative electrostatic interactions. Such systems are capable of specifically interacting with a third component (the drug being administered) [15,16].

As a cationic peptide antibiotic, CT has the ability to interact with anionic molecules, including anionic polymers, and to form negatively charged NPs, and the resulting complexes can be used as carriers for the targeted delivery of CT [6,17,18,19,20]. For example, our research group [21] studied the formation of CT delivery systems formed by complexation of the drug with HA via electrostatic interaction. The resulting particles had a negative surface charge (−19 mV) and ranged in size from 200 nm to 1 μm, depending on the ratio of HA/CT and the molecular weight (MW) of HA. The encapsulation efficiency (EE) was 30–100%, and the loading efficiency (LE) was 20–67%. The antibiotic release was 45% and 85% in 15 and 60 min, respectively (pH 7.4). The minimum inhibitory concentrations (MICs) of both encapsulated CT and pure CT were 1 μg/mL at pH 7.4 (against *Pseudomonas aeruginosa*). 

The positively charged CT can also be loaded into a system consisting of a polycation and a polyanion. For example, Yasar et al. [6] developed polyelectrolyte complexes (PECs) based on anionic starch (MW > 100,000) and oligochitosan (MW 5000) for the delivery of CT and tobramycin. These PECs had a size of 170–380 nm, and a surface charge from −17 mV to −30 mV; the EE was 97–99% and the LE was 17% (for CT) and 3% (for tobramycin). The drug release into phosphate-buffered saline (PBS) at pH 7.4 reached 20–40% in 16 h. The antimicrobial activity of the loaded antibiotics against *E. coli* and *P. aeruginosa* was similar to that of the corresponding free drugs. Deacon et al. [19] fabricated NPs of tobramycin with alginate and alginate/chitosan and found that the presence of low-molecular-weight chitosan increased the colloidal stability of the NPs. These NPs had the following parameters: a size of 500–540 nm, ζ-potential of −25 to −28 mV, EE of 20–45%, and a tobramycin release (PBS, pH 7.4) of 45% in the first 90 min, which increased to 80% after 48 h. The antimicrobial activity of the NPs against *P. aeruginosa* was equivalent to that of unencapsulated tobramycin (MIC 0.625 mg/L). Balmayor et al. [20] produced an injectable biodegradable starch/chitosan delivery depot system for the sustained release of gentamicin for the treatment of bone infections. The obtained microparticles, prepared by a reductive alkylation crosslinking method, had a spherical shape, a size range of 80–150 μm, EE of 50–60%, and LE of 30%. The in vitro release profile (PBS, pH 7.4) was 50–70% in 24 h, followed by a sustained release for 30 days. In addition, a bacterial inhibition test on *Staphylococcus aureus* showed 70–100% of the antimicrobial activity of free gentamicin.

In summary, the delivery of vital antibiotics (polypeptides, macrolides, aminoglycosides, and fluoroquinolones) can be improved by encapsulating these drugs in carriers based on polysaccharide PECs. In this case, the antibiotic release is regulated by modification of the polymer MW, the component ratio, the particle size, and/or the preparation method.

We previously studied polyelectrolyte interactions between HA and DEAECS [7] and showed that mixing HA (MW of 950,000) with DEAEC (degree of substitution (DS) of 26, 55, 85, and 113%) resulted in the formation of spherical NPs whose size and charge depended both on the ratio of the polymers and on the mixing order. The most stable PEC was obtained by mixing HA and DEAEC at ratios of 1:5 and 1.7:5. The hydrodynamic diameter of these particles was 120–300 nm, and their surface charge was −10 mV to −23 mV. The structure–sensitivity ratio (Rg/Rh), determined by comparing the gyration radius (Rg) obtained by static light scattering and the hydrodynamic radius (Rh) obtained by dynamic light scattering (DLS), depended on the DS of the DEAECS. The use of chitosan with a DS of 25% and 55% gave an Rg/Rh of 1.1, whereas chitosan with high DS (85% and 113%) gave an Rg/Rh of 0.7. Spherically symmetric objects have an Rg/Rh of about 1, while monodisperse spheres have values of 0.7–0.8.

DEAECS [22,23,24,25] and HA [26,27,28,29] have attractive biomedical properties, as both are biodegradable, biocompatible, and non-toxic, water-soluble polymers. Chitosan and its water-soluble derivatives are enzymatically biodegraded in body fluids and tissues by lysozyme and are mainly distributed in the kidneys and urine, where they are further destructed by enzymes and excreted; this prevents their accumulation in the body [30,31,32]. HA has a high affinity for stabilin-2 and CD44 receptors (expressed at inflammation sites and on the surface of immunocompetent cells, such as T- and B-lymphocytes and macrophages) [33,34,35,36,37,38] and for toll-like receptor 4 (TLR4), which is associated with protection against Gram-negative bacteria [39,40]. The aim of the present work was to develop a suitable drug delivery system for improved CT delivery based on HA-DEAECS PECs. The CT delivery systems were expected to show an initial burst release of CT to provide a therapeutic dose in the systemic circulation at 0.5–1 h, followed by a sustained release to maintain an essential dose and antimicrobial activity for at least 10–12 h. 

We determined the optimal conditions for the formation of suitable complexes by studying parameters that affect the particle size and surface charge, as well as the EE and the LE. The parameters included: (i) the MW of HA, (ii) the mass ratio of HA and CT, and (iii) the addition of strong polycation DEAECS. We hypothesized that the inclusion of CT in the HA-based polymer system would result in prolonged and targeted action due to the high affinity of HA for CD44 receptors, while DEAECS would increase the stability of the complex and allow controlled CT release. The advantages of these polymer carriers are expected to improve CT delivery and antimicrobial activity, while reducing CT side effects.

## 2. Results and Discussion

### 2.1. Preparation and Characterization of HA-DEAECS PECs

**Scheme 1:**
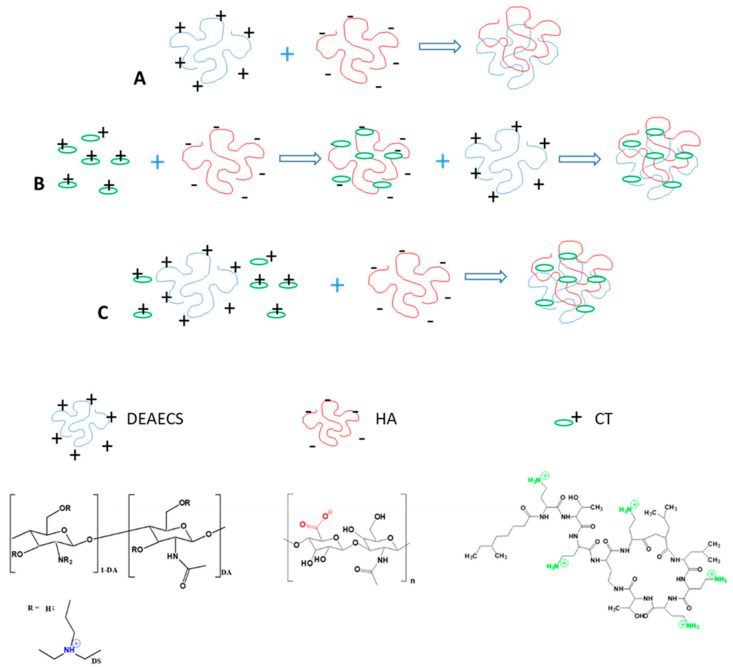
Preparation of HA-DEAECS PEC (**A**) and CT-HA-DEAECS PEC (**B**,**C**).

The first step in our study was the preparation and characterization of drug-free HA-DEAECS PECs (Scheme 1A). This step had two purposes: one was to study the effects of MW and DS on the characteristics of the resulting particles to allow later comparisons with CT-loaded NPs, and the other was to obtain drug-free particles for use in biological experiments to exclude the influence of NPs on antimicrobial activity and toxicity. We used polyanion HA with different MWs to have better control of the NP size, and we used polycation DEAECS with different DS to have better control of the strength of the formed PEC and the surface charge. Both HA and DEAECS are water-soluble, biocompatible, biodegradable, and non-toxic, and their potential to serve as drug delivery vehicles is enhanced by their simple preparation procedure. HA is an alternating copolymer of D-glucuronic acid and N-acetyl-D-glucosamine, linked via alternating β-(1→4) and β-(1→3) glycosidic bonds. DEAECS is an alkylated derivative of chitosan. As shown previously, both amine and hydroxyl groups are substituted, and 0–15% of the diethylaminoethyl groups are alkylated, forming quaternary ammonium groups [7].

The hydrodynamic diameter and ζ-potential of the resulting PEC are presented in Table 1. The HA-DEAECS PECs were obtained with a negative surface charge, as evidenced by a ζ-potential of around −32 to −35 mV. The hydrodynamic diameter of the PECs varied from 400 to 2000 nm, and this depended heavily on the MW of the HA. The obtained data led to the choice of HA_54_ for further experiments. 

**Figure 1:**
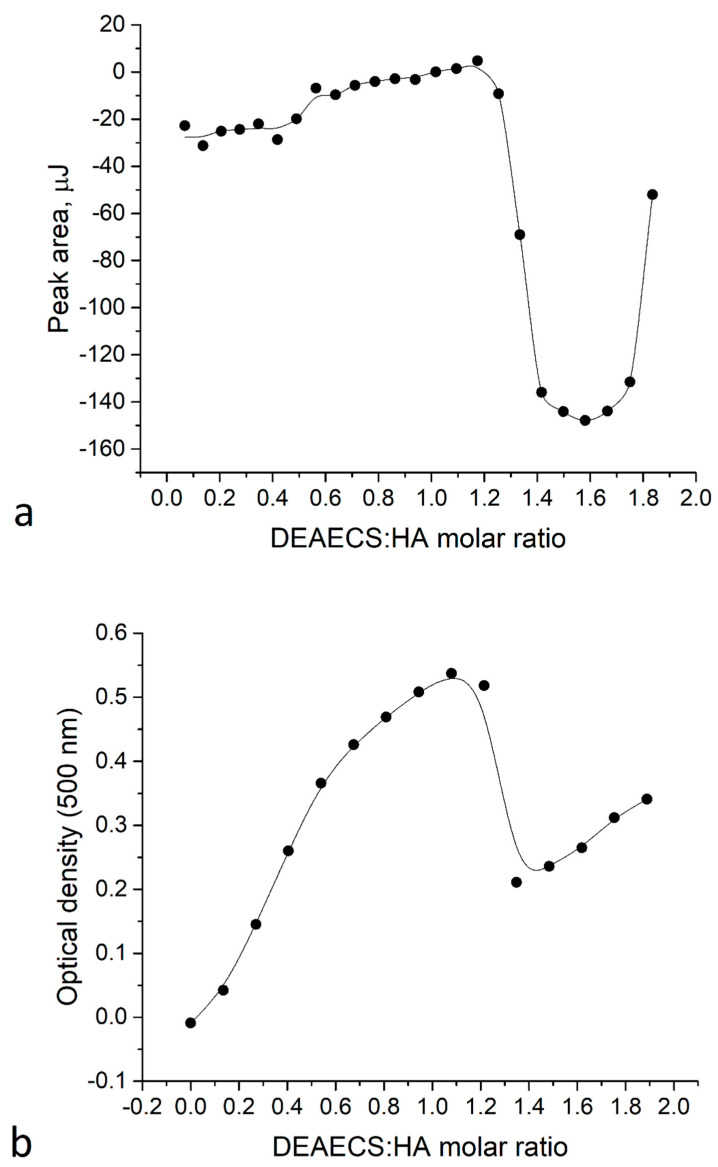
(**a**) Isothermal titration calorimetry (ITC) titration curve; (**b**) Turbidimetry data for HA_54_ titration by DEAECS_84_.

The thermodynamic stability of the HA-DEAECS PECs was studied using isothermal titration calorimetry (ITC). The ITC data (Figure 1) are represented by two fragments: the first is the PEC formation with an endothermal effect. Points for molar ratios from 0 to 1 were fitted by a one-site independent binding model, and the corresponding thermodynamic parameters were Kd = 3.49 × 10^−5^; n = 0.521; ΔH = −0.207 kJ/mol; ΔG = −25.44 kJ/mol; and ΔS = 84.64 J/mol×K. Therefore, not surprisingly, the HA-DEAECS PEC formation is an entropy-driven process. 

The heat effect at a molar ratio of 1.2–2 could not be explained by the ionic interactions of HA and DEAECS. Turbidimetry data (Figure 1b) showed that a molar ratio of 1.2–1.4 led to a rapid decrease in turbidity. This reflects an endothermic effect on the ITC curve and suggests that this effect represents a disaggregation at an excessive amount of titrant. 

Consequently, the HA-DEAECS PEC preparation procedure represents a simple method for the formulation of stable anionic particles of different sizes.

### 2.2. Preparation, Optimization, and Characterization of CT-HA-DEAECS PECs

CT is a cyclic heptapeptide with a tripeptide side chain that bears the covalently attached fatty acid. It is positively charged at a neutral pH due to the presence of amine functional groups in the structure. The positively charged CT molecules are able to interact with polyanionic HA molecules and form PECs. In addition, the CT molecule contains a lipophilic fatty acyl tail and a hydrophilic head group; therefore, CT can form micellar structures with anionic polymers that are further stabilized by the addition of chitosan and chitosan derivatives [6,17,21]. The use of this type of polyelectrolyte particle as a systemic circulation drug carrier requires that the particles have a size below 300 nm and a negative charge below −15 mV to ensure stability. We investigated the possibility of using HA and DEAECS to load CT by studying the influence of various factors on the size, ζ-potential, EE, and LE. The obtained results are presented in Table 1. 

One point to note is that the introduction of CT into the HA-DEAECS (5:1) PECs reduced their size from 400–600 nm to 210–244 nm (HA_54_) and from 1800 to 600 nm (HA_750_), regardless of the DS of DEAECS. These results likely reflect the high efficiency of CT binding to HA. The ζ-potential of the CT-loaded PEC decreased from −(31–32) mV to −(18–19) mV (HA_54_) and from −35 mV to −24 mV (HA_750_) compared to CT-free particles.

Further experiments were aimed at examining the effect of DEAECS addition on the properties of the resulted PEC. As presented in Table 1, the addition of DEAECS did not significantly change the particle size. The surface charge was significantly reduced only for the CT:HA:DEAECS ratios of 2:5:1 and 4:5:1. We used the CT:HA:DEAECS ratio of 1:5:1 to study the effect of DS, the amount of DEAECS_84_, and the order of addition of the components to the HA. The obtained data show that these factors did not affect the particle size, whereas a 2-fold increase in the DEAECS_84_ amount in the CT-HA-DEAECS PEC changed the ζ-potential from −19 to −15 mV. The addition of DEAECS_84_ to the CT-HA nanosuspension changed the ζ-potential from −19 to −13 mV. The EE and LE for CT-HA-DEAECS_84_ depended primarily on the amount of the loaded CT; the best values were 100% (EE) and 16.7% (LE) for the CT:HA:DEAECS ratio of 1:5:1 (Table 1). The PDI values for most formulations were in the range of 0.1–0.2; PDI values of 0.2 and below are generally considered acceptable for nanocarrier drug delivery systems [41].

Based on the optimization results, formulations 5, 10, 11, 12, and 13 were selected for further study to test the stabilizing effect of DEAECS on CT release and on CT antimicrobial activity.

**Figure 2:**
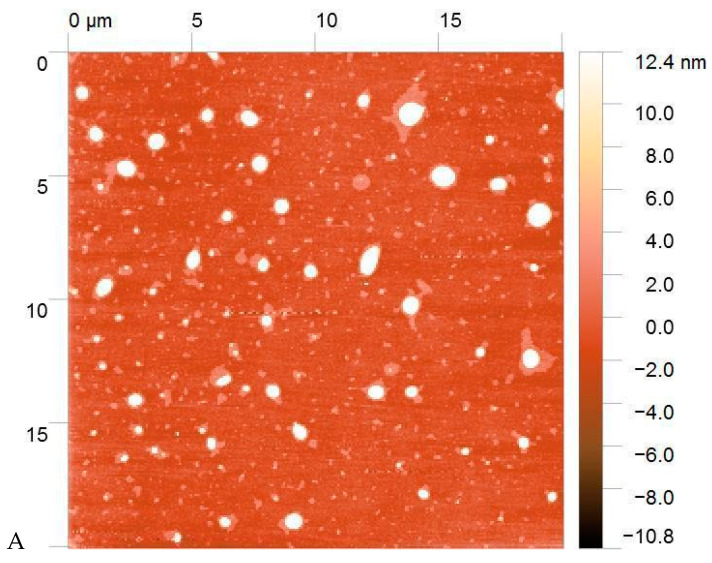

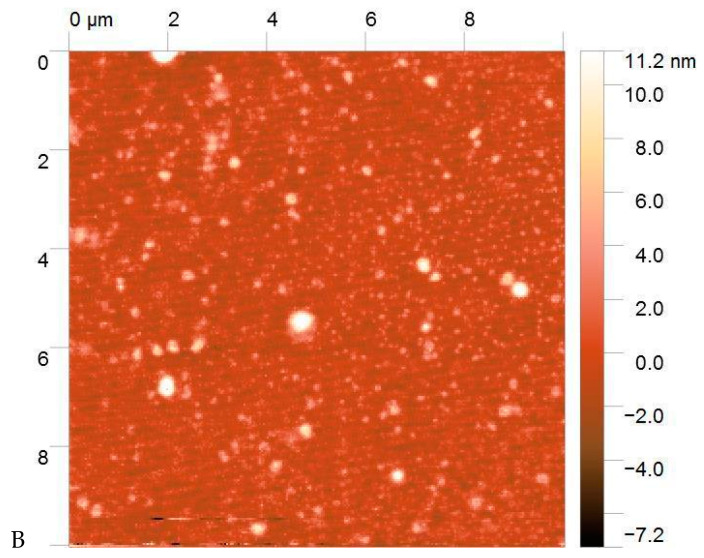
Atomic force microscopy (AFM) images of Formulation 1 (**A**) and Formulation 5 (**B**).

The morphology of the PECs was visualized using AFM (Figure 2). The resulting particles had a spherical shape with a size ranging from 10 to 500 nm, with most at 50–100 nm. The Formulation 1 particles were surrounded by non-complexed macromolecules. The particle size corresponds to the size estimated using DLS, taking into consideration that AFM measurements were carried out on dry samples that reduced the particle size compared to the hydrated complexes in suspension. 

### 2.3. In Vitro Release of CT from CT-HA-DEAECS PECs

**Figure 3:**
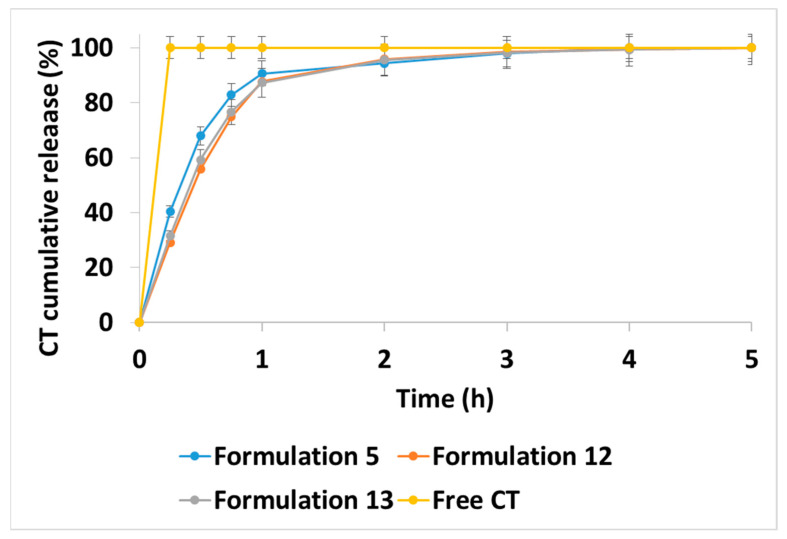
Colistin (CT) release kinetics from different CT-HA-DEAECS PECs formulations at 37 °C into phosphate-buffered saline (PBS). Free CT corresponds to an equivalent concentration of the CT in the PEC. Error bars indicate standard deviations (n = 3).

The study of release kinetics into PBS (pH 7.4) simulated the pattern of CT behavior in the systemic bloodstream. As shown in Figure 3, the CT release was 30–40% in 0.25 h, with 85–90% of the CT being released in 1–2 h for all three tested formulations (formulations 5, 12 and 13). Thus, when loaded into the HA-DEAECS PEC via polyelectrolyte interactions, CT was rapidly released due to the high ionic strength of the release medium. A 2-fold increase in DEAECS amount (formulations 12 and 13) stabilized the PEC and slowed this CT release, but for only 1 h. After the first hour, the release from all test samples was approximately the same [6,19].

The proposed modification of CT release from polymeric PEC compared to the release profile of free CT will allow targeted drug delivery to the sites of bacterial inflammation due to the high affinity of HA for the relevant receptors (CD44, stabilin-2, and TLR4 [33,34,35,36,37,38,39,40]. However, the initial rapid CT release is also necessary to ensure high antibiotic doses in the first hours after administration, since the bactericidal effect of CT is mainly realized via the carpet model of insertion, and it is dose-dependent [42,43,44,45]. 

### 2.4. Antimicrobial Activity of CT-HA-DEAECS PECs

**Figure 4:**
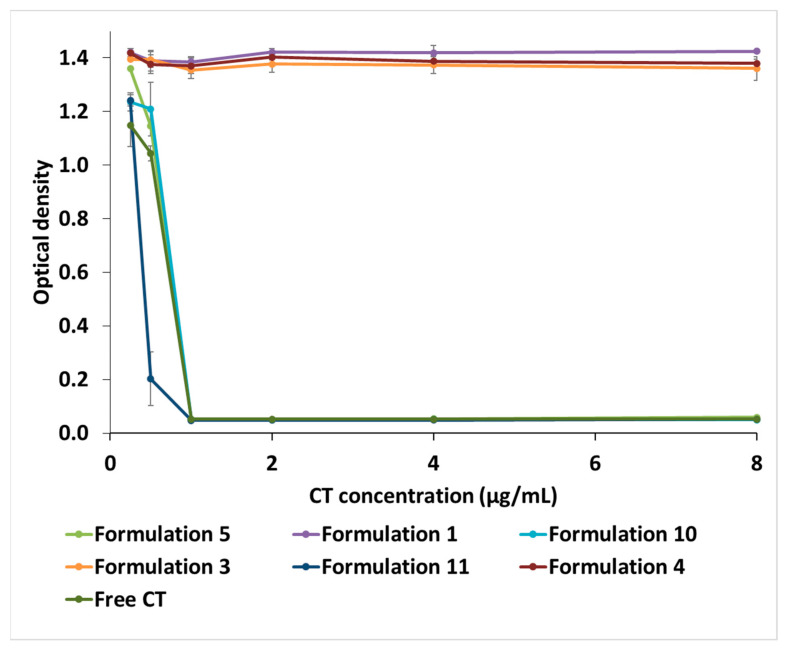
The minimum inhibitory concentrations (MIC) of CT-HA-DEAECS and HA-DEAECS PECs against *Pseudomonas aeruginosa.* Error bars indicate standard deviations (n = 3).

The MIC for *Pseudomonas aeruginosa* was determined at a concentration of 1 × 10^7^ CFU/mL (Figure 4). The CT-loaded NPs showed comparable antimicrobial effects to those of unencapsulated CT. All MICs were 1 μg/mL, which may indicate the preservation of the antibiotic activity in spite of encapsulation in different types of NPs. The blank NPs had no discernible effects on the visible growth of the bacteria.

The in vivo test showed that the encapsulated CT was released after 1–2 h in PBS, but it could present different kinetics in the microbial culture medium. Therefore, the study of the antimicrobial activity of developed drug delivery systems confirms that CT remains active and that encapsulated CT is released at a sufficiently rapid rate to maintain the 24 h result.

## 3. Materials and Methods

### 3.1. Materials 

We used HA with MWs of 54,000 (HA_54_) and 750,000 (HA_750_). The MWs were determined by viscometry. The intrinsic viscosity of HA was determined using an Ubbelohde viscometer (Design Bureau Pushchino, Pushchino, Russia) at 30 °C in 0.2 M NaCl. The MW of HA was calculated from the Mark–Houwink equation: [η] = 3.9 × 10^−2^ × MW^0.77^; [η] HA_54_ = 1.7 dL/g and [η] HA_750_ = 13.1 dL/g [46,47]. 

DEAECS with different DS values of 45% (DEAECS_45_), 64% (DEAECS_64_), and 84% (DEAECS_84_), determined by ^1^H NMR spectroscopy [7], were synthesized using a previously described method [7]. The starting material was crab shell chitosan with an average MW of 37,000 (determined by viscometry) and a degree of acetylation (DA) of 26% (determined by elemental analysis and ^1^H NMR spectroscopy) [48].

CT sulfate (MW of 1390) was procured from BetaPharm (Wujiang, Shanghai, China). The contents of CT A (polymyxin E1) and CT B (polymyxin E2) were 31.1 ± 0.4% and 68.9 ± 0.4%, respectively [49]. 

2-Chloro-*N*,*N*-diethylamine hydrochloride (99%), deuterium oxide (99.9 atom % D), trifluoroacetic acid, and PBS were obtained from Sigma-Aldrich Co. (St. Louis, MO, USA). Other reagents and solvents were obtained from commercial sources and were used without further purification. 

### 3.2. General Methods

The ^1^H NMR spectra were recorded on an Avance 400 spectrometer (Bruker BioSpin GmbH, Rheinstetten, Germany) at 400 MHz and 70 °C using a zgpr pulse sequence with suppression of residual H_2_O. Samples for NMR were dissolved in D_2_O and X μL of CF_3_COOH (where X is the mass of the polymer) was added to protonate all the amino groups.

Elemental analysis was performed on a Vario EL CHN analyzer (Elementar, Langenselbold, Germany). 

The hydrodynamic diameter (2Rh) and the ζ-potential were determined by dynamic and electrophoretic light scattering, respectively, using a Compact-Z instrument (Photocor, Moscow, Russia) with a 659.7 nm He–Ne laser at 25 mV power and a detection angle of 90°. The polydispersity index (PDI) was determined by cumulants’ analysis of autocorrelation function using DynaLS software. 

The particle morphology was studied using atomic force microscopy with a Smena instrument (NT-MDT, Zelenograd, Russia).

### 3.3. Liquid Chromatography-Mass Spectrometry (LC-MS) Measurements

LC-MS was performed as previously described [21]. Chromatographic separation was performed using an Elute UHPLC (Bruker Daltonics GmbH, Bremen, Germany) equipped with a Millipore Chromolith Performance/PR-18e, C18 analytical column (100 mm × 2 mm, Merck, Darmstadt, Germany) with Chromolith^®^ RP-18 endcapped 5-3 guard cartridges (Merck, Darmstadt, Germany). Mass spectra were obtained on a Maxis Impact Q-TOF mass spectrometer (Bruker Daltonics GmbH, Bremen, Germany) equipped with an electrospray ionization source (Bruker Daltonics GmbH, Bremen, Germany) and operated in positive ionization mode. Mass spectra were analyzed using DataAnalysis^®^ and TASQ^®^ software (Bruker Daltonics GmbH, Bremen, Germany). 

### 3.4. Isothermal Titration Calorimetry and Turbidimetry 

HA_54_ (2.12 mM; moles of monomeric units) and DEAECS_84_ (14.3 mM; moles of N atoms) solutions in 0.15 M NaCl were prepared and degassed under vacuum before the titration. HA_54_ (1 mL) was loaded in a Nano ITC microcalorimeter (TA Instruments, New Castle, DE, USA) and titrated by 24 injections (9.98 µL) of DEAECS_84_ solution. The time interval between injections was 2400 s, and the temperature was 25.0000 ± 0.0001 °C. ITC measurements were performed at the Center for Thermogravimetric and Calorimetric Research of Science Park of St. Petersburg State University.

In parallel, 2 mL of 2.12 mM HA_54_ solution were loaded into a 10 mm quartz cuvette and titrated with 14.3 mM DEAECS_84_ solution. After each 20 µL addition, the optical density (OD) at 500 nm was measured on a UV-1700 spectrometer (Shimadzu, Kyoto, Japan). 

### 3.5. Preparation of HA-DEAECS PECs

Solutions of HA (0.5 mg/mL, pH 6.5) and DEAECS (0.5 mg/mL, pH 5.6–6.2) with a DS of 45%, 64%, and 84% were prepared in ultrapure water.

Method A: HA-DEAECS PECs were obtained by the following procedure [7]: DEAECS solution was added dropwise via a 23G needle into the HA solution (Scheme 1A) at a ratio of 1:5, and the mixture was vortexed for 2 min and then allowed to stand for 2 h for PEC formation. Various PEC formation parameters are presented in Table 1. The resulting NPs were freeze-dried. 

### 3.6. Preparation of CT-HA-DEAECS PECs

Solutions of CT (1 mg/mL, pH 6.9), HA (0.5 mg/mL, pH 6.5), and DEAECS (0.5 mg/mL, pH 5.6–6.2) with DS of 45%, 64%, and 84% were prepared in ultrapure water. CT-HA-DEAECS PECs were obtained by the following procedures. 

Method B: CT solution was added to the HA solution, and the DEAECS solution was added to this mixture (Scheme 1B). 

Method C: CT was mixed with the DEAECS solution, and this mixture was added to the HA solution (Scheme 1C). All solutions were added dropwise via a 23G needle, and the mixtures were vortexed for 2 min and then allowed to stand for 2 h for forming PECs. Various PEC formation parameters are presented in Table 1. The resulting NPs were freeze-dried.

### 3.7. Encapsulation and Loading Efficiencies

The EE and LE were determined by measuring the concentration of the non-loaded CT (by an indirect method). The CT-HA-DEAECS PEC suspension was concentrated by ultrafiltration at 4500 rpm through a 10,000 MWCO Vivaspin^®^ Turbo 4 centrifugal concentrator (Sartorius AG, Göttingen, Germany) for 15 min at 20 °C. The amount of encapsulated CT in the PEC was calculated by the difference between the total CT amount used to prepare the PEC and the CT amount in the supernatant. The CT concentration in the supernatant was analyzed by LC-MS. The results were calculated using the following equations:

$$\mathrm{EE}\;(\%)=\frac{\mathrm{CT}\;\mathrm{mass}\;\mathrm{total}-\mathrm{CT}\;\mathrm{mass}\;\mathrm{in}\;\mathrm{the}\;\mathrm{supernatant}}{\mathrm{CT}\;\mathrm{mass}\;\mathrm{total}}\times 100\%$$

$$\mathrm{LE}\;(\%)=\frac{\mathrm{CT}\;\mathrm{mass}\;\mathrm{total}-\mathrm{CT}\;\mathrm{mass}\;\mathrm{in}\;\mathrm{the}\;\mathrm{supernatant}}{\mathrm{Polymer}\;\mathrm{mass}}\times 100\%$$

### 3.8. In Vitro Release of CT from CT-HA-DEAECS PEC 

The release medium conditions took into account the FDA recommendations for the dissolution methods of injectable dosage forms. A 1 mg sample of PEC was dissolved in PBS (4 mL, pH 7.4) and incubated at 37 °C. At regular intervals, 4 mL of medium was ultracentrifuged at 4500 rpm using a 10,000 MWCO Vivaspin^®^ Turbo 4 centrifugal concentrator. The amount of CT released into the supernatant was determined by LC-MS. 

### 3.9. Antimicrobial Activity of CT-HA-DEAECS PEC 

This assay was realized by the microtiter broth dilution method described by the Clinical and Laboratory Standards Institute [50] using *P. aeruginosa* ATCC 27,853 (Museum of Microbiological Cultures, State Research Institute of Highly Pure Biopreparations, St. Petersburg, Russia).

In a 96-well plate, 125 μL of Mueller-Hinton broth (HiMedia, Mumbai, India) were added. Stock solutions of NPs with antibiotics were prepared by diluting the sample in Mueller-Hinton broth to a maximum 2-fold required CT concentration. Serial dilutions of CT at concentrations from 64 to 0.25 μg/mL were then obtained on the plate. The OD of an overnight *P. aeruginosa* suspension in Mueller-Hinton broth was measured on a UVmini-1240 spectrophotometer (Shimadzu, Kyoto, Japan) at a wavelength of 540 nm, and the suspension was serially diluted 1:100 in Mueller-Hinton broth to give approximately 1 × 10^7^ CFU/mL. 

The inoculum was prepared by cultivation in a liquid medium (Mueller-Hinton broth) for 18 h by adding 125 μL of inoculum to the wells of the plate. The plate also contained controls: 100% growth (bacteria only), sterility control (Mueller-Hinton broth only), and blank NPs at equivalent polymer concentrations. The plate was incubated for 24 h at 37 °C, and then the OD was measured on an ELx808 ™ Absorbance Microplate Reader (BioTek Instruments, Winooski, VT, USA) at 630 nm.

## 4. Conclusions

This study has several main conclusions: (i) Polyanionic HA and polycationic DEAECS in aqueous solutions form stable PECs that are suitable for the inclusion of positively charged CT molecules. The reaction entropy component (−TΔS) and free energy (ΔG) were negative, indicating a favorable reaction. A negative ΔG corresponds to an entropy-driven process, which indicates strong bonding between the molecules; the Kd in the physiological solution was about 3.49 × 10^−5^. (ii) Varying the MW of HA, component ratios, and mixing procedure optimized the PEC preparation method and yielded formulations suitable for intravascular injection (mean size 210–250 nm and surface charge −19 mV). (iii) In vitro release experiments showed that the developed drug delivery systems provided sustained CT release for approximately 1–2 h compared to pure CT. (iv) The drug delivery systems exhibited comparable antimicrobial activity against *P. aeruginosa* to that of pure CT (both had a MIC of 1 µg/mL). This study can be extended to include in vivo biological tests to develop safe and effective antimicrobials for the treatment of infections caused by Gram-negative multidrug-resistant microorganisms.

## Figures and Tables

**Table 1:** Formation parameters and properties of HA-DEAECS and CT-HA-DEAECS PECs (mean ± standard deviation, n = 3).

No.	Formulation (Weight Ratio)	Procedure	2Rh (nm)	ζ-Potential (mV)	PDI	EE (%)	LE (%)
Formulation 1	HA_54_:DEAECS_84_ (5:1)	Scheme 1A	446 ± 136	−32.8 ± 0.2	0.11	-	-
Formulation 2	HA_750_:DEAECS_84_ (5:1)		1862 ± 442	−35.0 ± 0.8	0.09	-	-
Formulation 3	HA_54_:DEAECS_45_ (5:1)		604 ± 158	−31.7 ± 0.6	0.10	-	-
Formulation 4	HA_54_:DEAECS_64_ (5:1)		520 ± 76	−32.0 ± 0.6	0.06	-	-
Formulation 5	CT:HA_54_:DEAECS_84_ (1:5:1)	Scheme 1C	244 ± 66	−18.8 ± 0.2	0.10	100	16.7
Formulation 6	CT:HA_54_:DEAECS_84_ (2:5:1)		284 ± 134	−12.2 ± 0.6	0.24	98.5	32.8
Formulation 7	CT:HA_54_:DEAECS_84_ (4:5:1)		950 ± 248	−2.6 ± 0.2	0.10	47.8	31.8
Formulation 8	CT:HA_54_:DEAECS_84_ (6:5:1)		1104 ± 300	−1.9 ± 0.9	0.10	36.9	36.9
Formulation 9	CT:HA_750_:DEAECS_84_ (1:5:1)		612 ± 104	−24.4 ± 0.4	0.07	100	16.7
Formulation 10	CT:HA_54_:DEAECS_45_ (1:5:1)		210 ± 72	−19.0 ± 0.2	0.14	100	16.7
Formulation 11	CT:HA_54_:DEAECS_64_ (1:5:1)		210 ± 96	−18.9 ± 0.9	0.23	100	16.7
Formulation 12	CT:HA_54_:DEAECS_84_ (1:5:2)		210 ± 38	−14.9 ± 0.6	0.07	100	14.3
Formulation 13	CT:HA_54_:DEAECS_84_ (1:5:2)	Scheme 1B	210 ± 68	−13.5 ± 0.4	0.12	100	14.3

## Data Availability

The data are contained within the article.
